# CT-based radiomic signatures for prediction of pathologic complete response in esophageal squamous cell carcinoma after neoadjuvant chemoradiotherapy

**DOI:** 10.1093/jrr/rrz027

**Published:** 2019-05-21

**Authors:** Zhining Yang, Binghui He, Xinyu Zhuang, Xiaoying Gao, Dandan Wang, Mei Li, Zhixiong Lin, Ren Luo

**Affiliations:** 1 Department of Radiation Oncology, Cancer Hospital of Shantou University Medical College, 7 Raoping Road, Shantou, Guangdong, China; 2 Department of Radiation Oncology, Donghua Hospital Affiliated to Zhongshan University,1 Dongcheng East Road, Dongguan, Guangdong, China; 3 Eye Center, Medical Center—University of Freiburg, Killianstraße, Freiburg Germany; 4 Department of Radiation Oncology, Medical Center—University of Freiburg, Robert-Koch-Str. 3, Freiburg, Germany; 5 Faculty of Biology, University of Freiburg, Freiburg, Germany

**Keywords:** esophageal squamous cell carcinoma, neoadjuvant chemoradiotherapy, complete pathologic response, radiomics, LASSO

## Abstract

The objective of this study was to build models to predict complete pathologic response (pCR) after neoadjuvant chemoradiotherapy (nCRT) in esophageal squamous cell carcinoma (ESCC) patients using radiomic features. A total of 55 consecutive patients pathologically diagnosed as having ESCC were included in this study. Patients were divided into a training cohort (44 patients) and a testing cohort (11 patients). The logistic regression analysis using likelihood ratio forward selection was performed to select the predictive clinical parameters for pCR, and the least absolute shrinkage and selection operator (LASSO) with logistic regression to select radiomic predictors in the training cohort. Model performance in the training and testing groups was evaluated using the area under the receiver operating characteristic curves (AUC). The multivariate logistic regression analysis identified no clinical predictors for pCR. Thus, only radiomic features selected by LASSO were used to build prediction models. Three logistic regression models for pCR prediction were developed in the training cohort, and they were able to predict pCR well in both the training (AUC, 0.84–0.86) and the testing cohorts (AUC, 0.71–0.79). There were no differences between these AUCs. We developed three predictive models for pCR after nCRT using radiomic parameters and they demonstrated good model performance.

## INTRODUCTION

Esophageal carcinoma (EC) is the one of the most common malignancies, and it ranks sixth as a cause of cancer-related mortality globally [1]. Approximately 80% of the new cases occur in less developed regions in the world, and 60% of these cases occur in China [2]. Despite multimodality advances in treatment, it remains a devastating disease, with a 5-year overall survival rate of 15–25%. One of the main reasons for the poor prognosis of EC is that patients are often diagnosed at an advanced stage. To reduce the difficulty of operating and to improve outcomes, applying concurrent chemoradiotherapy before surgery is now the first-line option for locoregional disease. Patients having a pathologic complete response (pCR) after neoadjuvant chemoradiotherapy (nCRT) appear to have superior overall survival [3, 4]. On the other hand, non-responders to nCRT may receive no benefit from this therapy [5]. We urgently need to be able to identify the EC patients who will benefit from nCRT so that the best treatment can be given to each individual patient.

Radiomics is an emerging method that extracts quantitative features from computed tomography (CT) images, magnetic resonance (MR) images, positron emission tomography (PET) images, etc. Radiomics could be applied to identify tumor phenotype characteristics [6, 7], and to discover prognostic or predictive biomarkers for cancers [8, 9].

As well, radiomics can be applied in prediction of treatment response, e.g. pCR in EC after nCRT. Yip *et al.* studied the possibility of using CT-based texture features to predict tumor regression grade (TRG, 1–3 vs 4–5, Mandard *et al.* [10]) in a cohort of 31 EC patients [which included only 9 esophageal squamous cell carcinoma (ESCC)] patients after nCRT [11]. The pre- and post-treatment standard deviation of the histogram was significantly associated with TRG. However, those researchers only analyzed a small number (*n* = 6) of histogram-based texture features. Zhen *et al.* analyzed 214 CT-based radiomic features to predict pCR in 49 ESCC patients. Prediction models developed by support vector machine (SVM) or artificial neural network (ANN) algorithms can discriminate non-responders from responders [12]. The area under the receiver operating characteristic curve (AUC) is 0.818 in SVM and 0.927 in ANN algorithms. More studies have linked the radiomic features from 18-F-deoxyglucose (^18^F-FDG) PET scans and pCR in EC patients after nCRT. Combining clinical factors and ^18^F-FDG PET–based radiomic features improved the prediction ability [13]. Roelof *et al.* developed a prediction model combining clinical T-stage and restaging after nCRT, which can provide high discriminatory accuracy in predicting pCR (AUC, 0.81) [14].

However, most radiomic studies [11, 14–18] included both adenocarcinoma and squamous cell carcinoma patients. In contrast to patients with adenocarcinoma, patients with ESCC may have a higher pCR rate after nCRT [19, 20]. In pCR prediction analysis, grouping these two histological types of EC together may have a negative impact on the accuracy of the analytical results. Here, we used quantitative features from radiomics as prognostic biomarkers to build models to predict pCR for patients with ESCC after nCRT. This model may help doctors to make the the best clinical decision at the beginning of treatment.

## MATERIALS AND METHODS

### Patients

A total of 55 consecutive patients pathologically diagnosed as having ESCC were included in this retrospective study. The nCRT was administrated to all patients between May 2012 to August 2016 at the Cancer Hospital of Shantou University Medical College. Informed consent was obtained from all individual participants included in the study. All patients received a pre-treatment CT scan for radiotherapy planning.

The patients’ clinical stage of ECSS was performed according to the 7th edition of the American Joint Committee on Cancer (AJCC) staging system with CT scan and endoscopic ultrasonography. More information about the patients is listed in Table 1.

**Table 1.** Patient characteristics and the correlation between clinical factors and complete pathologic response after neoadjuvant chemoradiotherapy

	Training group		Multivariate logistic regression	Testing group		Differences between training and testing groups
Variables	*n* (%)	median (range)	*P*-value	*n* (%)	median (range)	*P*-value
Total patients	44 (80)			11 (20)		
pCR			NA			0.478^a^
Yes	19 (43)			4 (36)		
No	25 (57)			7 (64)		
Gender			0.504			0.449^a^
Male	35			8		
Female	9			3		
Age (years)		56 (32–68)	0.984		57 (49–66)	0.368^b^
Tumor length (cm)		7 (3.5–16)	0.910		7.5 (4–11)	0.585^b^
Tumor location			0.817			0.285^a^
Upper	10			4		
Middle or lower	34			7		
T-stage			0.176			0.577^a^
T3	19			5		
T4a	25			6		
N-stage			0.792			0.888^c^
N0	6			1		
N1	33			9		
N2	5			1		
Chemotherapy regimen			0.528			0.782^c^
PF	19			6		
NP	19			4		
TP	6			1		
Technique			0.515			0.473^a^
3D-CRT	23			5		
IMRT	21			6		
Radiation dose (Gy)			0.251			0.685^a^
<50	36			9		
≥50	8			2		

pCR = pathologic complete response, NA = not available; PF = cisplatin + fluorouracil, NP = vinorelbine + cisplatin, TP = paclitaxel + cisplatin, 3D-CRT = 3D conformal radiotherapy, IMRT = intensity-modulated radiotherapy.

^a^Chi-squared test, ^b^independent *t* test, ^c^Fisher’s test

### Neoadjuvant chemoradiotherapy

Patients were treated with 3D conformal radiotherapy (3D-CRT) or intensity-modulated radiotherapy (IMRT) using 6 MV X-rays. The gross tumor volume (GTV) was identified using both diagnostic and radiotherapy planning CT images and barium esophagography, and GTV included the primary tumor (GTVp) and grossly involved regional lymph nodes (GTVn). The clinical target volume (CTV) was defined as the GTVp with a margin of 1.0 cm laterally and a 3.0 cm margin in the superior and inferior dimensions plus GTVn with a 0.5 cm to 1.5 cm expansion. The PTV was determined by adding 0.5 cm radially to the CTV. A total prescription dose of 40–64 Gy (median, 50 Gy) was delivered in 2 Gy per fraction 5 days a week.

A concurrent 3-weekly schedule of platinum-based nCRT was administrated to all patients. Twenty-three patients received NP (vinorelbine + cisplatin) chemotherapy, which consists of cisplatin (75 mg/m^2^ on Day 1) plus vinorelbine (25 mg/m^2^ on Days 1 and 8). Twenty-five patients received the PF (cisplatin + fluorouracil) regimen, which consists of cisplatin (75 mg/m^2^ on Day 1) and fluorouracil (750 mg/m^2^/24 h on Days 1 to 4). For the TP (paclitaxel + cisplatin) regimen for 7 patients, paclitaxel was administrated using 135–180 mg/m^2^ on Day 1 and cisplatin 75 mg/m^2^ on Day 1. In cases of severe hematologic toxicity, dose adjustment was implemented in the second chemotherapy cycle.

### Surgery

All patients underwent clinical re-examination 4 weeks after nCRT, including a barium esophagography test and thoracoabdominal CT. A transthoracic esophagectomy with two-field or three-field lymphadenectomy was performed 5–6 weeks after the neoadjuvant treatment. A pCR patient was defined as a patient with no residual, viable tumor cells in the surgical specimen.

### CT image radiomic feature collection

For all patients, CT scans (CT scanner: Philips Brilliance CT Big Bore Oncology Configuration, Cleveland, OH) were performed in the supine position with intravenous contrast. A standard clinical acquisition protocol (tube voltage, 120 kVp; rotation time, 0.75 seconds; pitch, 0.938; matrix, 512 × 512; field of view, 350 mm × 350 mm; pixel size, 1.46 mm; slice thickness, 5 mm; reconstruction kernel, standard) was applied for each patient in this cohort. No resample of the voxel size of the CT images was used. The bit depth of patients’ CT images was 12 and the number of gray levels was 4096. The GTVs of ESCC were delineated for the ESCC on the planning non-enhanced CT-scan by experienced radiation oncologists, using a reference of barium radiography of the esophagus or contrast-enhanced CT. A 3DSlicer (version, 4.8.1, Stable Release) with its extension (radiomics) was used for collecting the radiomic features from pre-treatment CT [21]. Any pixel with an attenuation of less than −50 HU was excluded to remove the intra-luminal air from GTVs. In image pre-processing, Laplacian of Gaussian or wavelet filters were used. Five values of Laplacian of Gaussian spatial band-pass filter (0, no filtration; 1.0, fine textures; 1.5 and 2.0, medium textures; 2.5, coarse textures) for image smoothing and a fixed number of three fixed number (32, 64 or 128) of discrete bins for image resampling were applied. In the wavelet filter, the Coiflet 1 mother wavelet was used and a high-pass filter or low-pass filter were applied in the *x*, *y* and *z* directions. In all, 624 wavelet features (Supplemental Table 1) and 406 non-wavelet features (Supplemental Table 2) in each bin size (=32, 64 or 128) were collected. Among 406 non-wavelet features, there were 16 shape features, 19 × 5 (19 classes of first-order feature with 5 values of Laplacian of Gaussian spatial band-pass filter) first order features, 27 × 5 Gray Level Co-occurrence Matrix (GLCM) features, 16 × 5 Gray Level Size Zone Matrix (GLSZM) features, and 16 × 5 Gray Level Run Length Matrix (GLRLM) features. These radiomic features have previously been described [21].

### Statistical analysis

The 55 patients were divided into two groups (a training group of 44 patients, and a testing group of 11 patients). We performed multivariate logistic regression analysis using likelihood ratio forward selection in the training group to select the most predictable clinical factors for pCR. All the radiomic features were normalized using *Z*-score normalization. Three groups of radiomic data were analyzed separately: Group 1, non-wavelet features with bin size = 32 and all the wavelet radiomic features; Group 2, non-wavelet features with bin size = 64 and all the wavelet radiomic features; Group 3, non-wavelet features with bin size = 128 and all the wavelet radiomic features. The least absolute shrinkage and selection operator (LASSO) with logistic regression was applied to select optimal predictors in the training group. LASSO with 10-fold cross-validation was performed using the *glmnet* [22, 23] package in R software (version 3.3.1, http://www.r-project.org/). Models based only on clinical predictors or the combination of clinical and radiomic signatures were built using logistic regression for pCR prediction.

Multivariable logistic regression formula:

$$P\left(S\right)={\left(1+e^{-\mathrm{bS}-c}\right)}^{-1},$$

where P is the probability of the event occurring; S = β_0_ + β_1_*x*_1_ + β_1_*x*_1_ + … + β_m_*x*_m_, where *x*_1_, *x*_2_ . . . *x*_m_ are different input parameters, β_0_ is the constant for S, and β_1_ ... β_m_ are the logistic regression coefficients of the corresponding input parameters. In this paper, S is the function for radiomic signature, b is the coefficient for S, and c is the constant in logistic regression.

Model performance was evaluated by the AUC using *pROC* [24] package in R software in both the training and testing groups. The AUCs were compared using the method suggested by Delong *et al.* [25] through *pROC*. The Chi-squared test or Fisher’s test was used to determine whether there was a significant difference in the categorical variables between these groups. A *P*-value of < 0.05 was considered statistically significant.

## RESULTS

The pCR rate of this study was 42% (23/55), 43% (19/44) and 36% (4/11) in the whole, training and testing cohorts after nCRT, respectively. No clinical differences were found between the training and testing groups (Table 1). No clinical factors were identified as predictable factors for pCR by logistic regression analysis in either the training or testing groups (Table 1). Three groups of radiomic features were analyzed by LASSO separately to build three radiomic signatures, and the results are presented in Table 2.

**Table 2.** Coefficients and features of three radiomic signatures for pCR

Coefficients	Features
Radiomic signature 1 (Sig 1)	
−0.283 (β_0_)	constant
−0.122 (β_1_)	bin32_original_shape_SurfaceVolumeRatio (*x*_1_)
−0.139 (β_2_)	bin32_log.sigma.2.5.mm.3D_firstorder_90Percentile (*x*_2_)
0.160 (β_3_)	bin32_wavelet.LLH_GLRLM_ZoneEntropy (*x*_3_)
0.181 (β_4_)	bin32_wavelet.HHH_GLRLM_RunEntropy (*x*_4_)
0.012 (β_5_)	bin32_wavelet.LLL_GLRLM_RunVariance (*x*_5_)
Radiomic signature 2 (Sig 2)	
−0.288 (β_0_)	constant
−0.014 (β_1_)	bin64_log.sigma.2.5.mm.3D_firstorder_90Percentile (*x*_1_)
−0.054 (β_2_)	bin64_wavelet.LHH_GLSZM_LowGrayLevelZoneEmphasis (*x*_2_)
0.068 (β_3_)	bin64_wavelet.LLH_GLSZM_ZoneEntropy (*x*_3_)
0.210 (β_4_)	bin64_wavelet.HLH_GLRLM_RunVariance (*x*_4_)
0.131 (β_5_)	bin64_wavelet.HHH_GLRLM_LongRunEmphasis (*x*_5_)
0.015 (β_6_)	bin64_wavelet.HHH_GLRLM_RunEntropy (*x*_6_)
Radiomic signature 3 (Sig 3)	
−0.312 (β_0_)	constant
−0.028 (β_1_)	bin128_log.sigma.2.5.mm.3D_firstorder_90Percentile (*x*_1_)
−0.143 (β_2_)	bin128_log.sigma.2.5.mm.3D_GLRLM_ShortRunLowGrayLevelEmphasis (*x*_2_)
0.247 (β_3_)	bin128_wavelet.HLH_GLRLM_RunVariance (*x*_3_)
0.116 (β_4_)	bin128_wavelet.HHH_GLRLM_LongRunEmphasis (*x*_4_)
−0.011 (β_5_)	bin128_wavelet.HHL_GLCM_ClusterProminence (*x*_5_)
−0.024 (β_6_)	bin128_wavelet.HHL_GLSZM_ZonePercentage (*x*_6_)

pCR = pathologic complete response, Filter “Wavelet”, H = high-pass filter (applied in the *x*, *y* and *z* directions, respectively), L = low-pass filter (applied in the *x*, *y* and *z* directions, respectively), pCR = pathologic complete response, GLCM = Gray Level Cooccurrence Matrix, GLSZM = Gray Level Size Zone Matrix, GLRLM = Gray Level Run Length Matrix.

**Figure 1.**
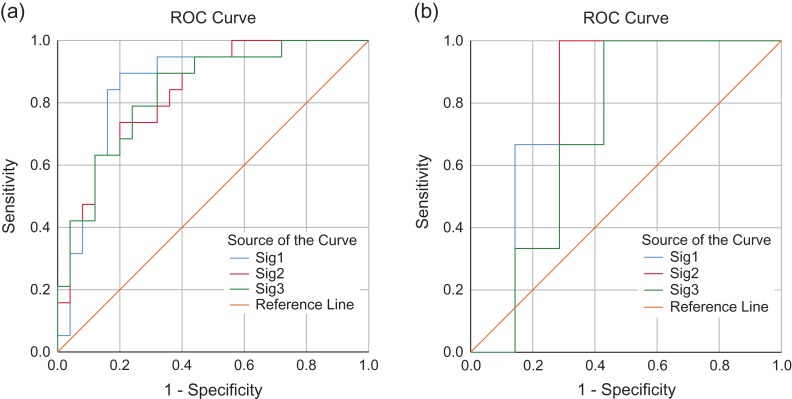
Comparison of receiver operator characteristic (ROC) curves obtained applying models (Model 1, 2 or 3) in training (Fig. 1a) and testing (Fig. 1b) groups.

Three logistic regression models for pCR prediction were developed based on these three signatures separately (Table 3). The AUCs of Model 1, Model 2 and Model 3 in the training dataset were 0.84 to 0.86 and in the testing group were 0.71 to 0.79 (Table 3). The receiver operating characteristic curves (ROCs) are shown in Fig. 1. There were no differences between these AUCs in the training group (Model 1 vs Model 2, *P* = 0.451; Model 1 vs Model 3, *P* = 0.483; Model 2 vs Model 3, *P* = 1.000) or in the testing group (Model 1 vs Model 2, *P* = 0.480; Model 1 vs Model 3, *P* = 0.401; Model 2 vs Model 3, *P* = 0.480).

**Table 3.** Coefficients, 95% confidence intervals and area under the receiver operating characteristic curves of three logistic regression models for pCR

Model	b	constant (c)	OR	95% CI	*P*-value	MSE	AUC in training group	AUC in testing group
Model 1		0.852					0.86 (95% CI, 0.74 to 0.98)	0.79 (95% CI, 0.48 to 1.00)
Sig 1	4.519		91.696	9.461–1900.114	0.001	0.996		
Model 2		0.891					0.84 (95% CI, 0.72 to 0.95)	0.75 (95% CI, 0.42 to 1.00)
Sig 2	4.538		93.483	8.571–2237.566	0.001	0.933		
Model 3		0.665					0.84 (95% CI, 0.72 to 0.96)	0.71 (95% CI, 0.38 to 1.00)
Sig 3	3.626		37.552	5.357–488.782	0.001	1.214		

pCR = pathologic complete response, b is the coefficient of corresponding radiomic signatures, OR = odds ratio, CI = confidence interval, MSE = mean squared error between training and testing cohort, AUC = the area under the receiver operating characteristic curve.

## DISCUSSION

Early identification of pCR prior to nCRT might avoid unnecessary chemoradiation-associated morbidity. However, there is still no powerful tool that can yield sufficient ability to predict pCR after nCRT [26, 27]. For example, using only ^18^F-FDG PET to predict pCR after nCRT in EC is not recommended [26]; not is applying the combination of ^18^F-FDG PET and endoscopic biopsy [27]. Radiomics may become a better method for predicting pCR. In previous studies that used radiomic data to predict pCR, the AUCs were between 0.71 and 0.93 [12, 14–18]. In the study that also analyzed CT-based radiomic features [12], the AUCs were the highest among these studies (0.818 in SVM and 0.927 in ANN modeling). However, modeling overfitting has likely occurred as a result of the small sample size (49 patients) and large number of predictors (214 radiomic features) included in the modeling. Although the number of patients in the present study was small (*n* = 55) as well, and more radiomic features (*n* = 1030) were analyzed in our study, the LASSO analysis was applied to handle overfitting in logistic regression and the AUCs were 0.84 to 0.86 in the training cohort and 0.71 to 0.79 in the testing cohort. Certainly, validating our models in an independent cohort is necessary before any clinical practice can be adopted, because of the small sample size and retrospective nature of our study.

In CT images, different scales of smoothing using Laplacian of Gaussian spatial band-pass filters are important; they can reduce image noise and highlight different anatomical spatial scales (from fine to medium to coarse texture) within the tumor [28, 29]. We applied five scales of smoothing (1 to 2.5, in steps of 0.5) to obtain the best radiomic features for pCR prediction, but only radiomic features with sigma = 2.5 were selected by LASSO. Therefore, the smoothing procedure using Laplacian of Gaussian spatial band-pass filters with suitable scales might be important in unenhanced CT images. Hatt *et al.* [30] found that significant texture details were lost when using a quantization of <32 bins. Based on the experience of PET-CT [30], we chose three different bins (32, 64 or 128) to resample the CT images. We found a similar prediction ability for the three radiomic signatures with the different bins. This finding is in line with previous findings claiming that ≥32 discrete values for the bin are recommended in order to properly quantify tumor heterogeneity [31], and that textural features computed with resampling values >64 may not provide additional prognostic information compared with the tumor volume [30]. However, although the features selected in all three models with similar performance appear to have been stable, the radiomic predictors differed between the three models. This suggests that we may need to use different bin sizes to resample the CT images and compare the performance of the different models for the different bin sizes. In these three models in our study, Model 1 had the highest AUC value (although the AUCs were not significantly different between the three models) in both the training and the testing cohorts, and it had the least number of predictors. Thus, when readers try to apply our models to their patients’ imaging, Model 1 might be the first choice.

Female sex, age, poor differentiation grade, tumor length, and low cT-stage were identified as the predictors for pCR after nCRT [20, 32, 33]. In our study, clinical factors were not found to be significantly related to pCR after nCRT, and this may be due to the relatively small number of ESCC patients. Although only radiomic features were selected, three radiomic signatures that we built could well predict the pCR in our cohort (AUCs, 0.71 to 0.80). Tumor volume is considered as a risk predictor for pCR [13, 30, 34]. However, the tumor volume failed to predict pCR (*P* = 0.157, AUC = 0.61, 95% CI, 0.46 to 0.76) in any of the patients in our cohort. The SurfaceVolumeRatio (bin = 32) (surface area to volume ratio, a lower value indicates a more compact shape; *P* = 0.04, AUC 0.66, 95% CI 0.52 to 0.81, for all patients) might provide more information about pCR than tumor volume. Hatt *et al.* found an added value of texture features over tumor volume alone for outcome prediction for tumors above 10 cm^3^ only [30]. However, the radiomic signatures provided better prediction performance in the case of only one patient with a tumor >10 cm^3^ in our cohort. Soufi *et al.* [35] tested wavelet radiomic features from different mother wavelets in survival prediction of non–small cell lung carcinoma patients; they found Symlet and Biorthogonal mother wavelets yielded the best performance. The radiomic tool that we used in this study only provides a wavelet radiomic feature from the Coiflet 1 mother wavelet. Thus, the wavelet radiomic features in our study might be not the optimal ones.

In the studies using texture features to predict pCR for EC [11, 14–18], both adenocarcinoma and squamous cell carcinoma patients were included, and the malignancy of the majority of these patients was adenocarcinoma. The AUCs in these ‘mixed’ studies were between 0.71 and 0.89. Compared with these ‘mixed’ studies, the AUCs in our study and that of Hou et al. [12], which both included only ESCC patients, seem to be higher (0.84–0.97). Hence, it might improve the model performance by dividing the patients according to histological types for different analyses in predicting pCR. The rationale behind this might be the differences in the pCR rate (49% in ESCC and 23% in adenocarcinoma) [19] and the genomic characterization [36]. Thus, including only one type of EC for analysis maybe more appropriate, and we developed this study to explore the radiomic predictors for ESCC.

## CONCLUSION

We developed three CT-based radiomic models for predicting the pCR in ESCC patients after nCRT. These predictive models demonstrated good model performance in predicting pCR and might help physicians identify candidates for nCRT.

## Supplementary Material

Supplementary Data

Supplementary Data
